# Effective hospital readmission prediction models using machine-learned features

**DOI:** 10.1186/s12913-022-08748-y

**Published:** 2022-11-24

**Authors:** Sacha Davis, Jin Zhang, Ilbin Lee, Mostafa Rezaei, Russell Greiner, Finlay A. McAlister, Raj Padwal

**Affiliations:** 1 Department of Computing Science, University of Alberta, Edmonton, AB Canada; 2 Alberta School of Business, University of Alberta, Edmonton, AB Canada; 3 Medicine and Dentistry, University of Alberta, Edmonton, AB Canada; 4 ESCP Business School, Paris, France; 5 Alberta Machine Intelligence Institute, Edmonton, AB Canada

**Keywords:** Hospitalization, Machine learning, Patient readmission, Area under curve

## Abstract

**Background:**

Hospital readmissions are one of the costliest challenges facing healthcare systems, but conventional models fail to predict readmissions well. Many existing models use exclusively manually-engineered features, which are labor intensive and dataset-specific. Our objective was to develop and evaluate models to predict hospital readmissions using derived features that are automatically generated from longitudinal data using machine learning techniques.

**Methods:**

We studied patients discharged from acute care facilities in 2015 and 2016 in Alberta, Canada, excluding those who were hospitalized to give birth or for a psychiatric condition. We used population-level linked administrative hospital data from 2011 to 2017 to train prediction models using both manually derived features and features generated automatically from observational data. The target value of interest was 30-day all-cause hospital readmissions, with the success of prediction measured using the area under the curve (AUC) statistic.

**Results:**

Data from 428,669 patients (62% female, 38% male, 27% 65 years or older) were used for training and evaluating models: 24,974 (5.83%) were readmitted within 30 days of discharge for any reason. Patients were more likely to be readmitted if they utilized hospital care more, had more physician office visits, had more prescriptions, had a chronic condition, or were 65 years old or older. The LACE readmission prediction model had an AUC of 0.66 ± 0.0064 while the machine learning model’s test set AUC was 0.83 ± 0.0045, based on learning a gradient boosting machine on a combination of machine-learned and manually-derived features.

**Conclusion:**

Applying a machine learning model to the computer-generated and manual features improved prediction accuracy over the LACE model and a model that used only manually-derived features. Our model can be used to identify high-risk patients, for whom targeted interventions may potentially prevent readmissions.

**Supplementary Information:**

The online version contains supplementary material available at 10.1186/s12913-022-08748-y.

## Introduction

### Background/rationale

Nearly 10% of patients hospitalized in Canada are readmitted within 30 days [1]. Readmissions cost approximately 2 billion Canadian dollars per year in Canada [2] in 2011 and 26 billion US dollars per year in the United States [3] in 2014. Studies estimate that 10–60% of these readmissions are avoidable [4–6]. In the US, the Centers for Medicare & Medicaid Services financially penalize hospitals with high readmission rates [7]. These consequences and costs of readmissions are one of the most important challenges facing the healthcare systems. Transitional care interventions may reduce readmissions, but these interventions are resource intensive. Predicting the readmission risk of individual patients can help better target these interventions, which can save expenses and may also suggest new ways to prevent readmissions.

Unfortunately, conventional models do not accurately predict readmissions; model c-statistics are rarely seen above 0.8 [8, 9]. Additionally, most of the existing prediction models rely heavily on manual feature engineering [5, 10–24], which is based on domain knowledge and experience. Those features are often dataset-dependent, thus limiting generalizability between datasets or jurisdictions. Recently, machine learning methods that automatically identify which parts of a given set of data are essential for prediction have gained popularity, and there exists such work applied in the domain of readmission prediction as well. Notably, Rajkomar et al. used electronic health records and deep learning models to predict 30-day readmissions and other outcomes [19]. However, their c-statistic for 30-day readmissions did not exceed 0.75 despite their c-statistics for other outcomes such as mortality being above 0.8. There have been several similar studies, but their c-statistics are also moderate, below 0.8 [19, 25, 26]. Choi et al. explored word embeddings to represent medical concepts [27–29], often paired with recurrent neural networks for the prediction of clinical events. This approach performed adequately on disease-specific tasks (e.g., heart-failure prediction, differential diagnosis), but they did not apply these techniques to hospital readmission prediction. Nguyen et al. [30] used similar techniques for hospital readmission but their target outcome was 3- and 9-month readmission and are thus not directly comparable.

### Objectives

This paper describes models to predict 30-day readmissions, with a focus on testing the predictive performance of input features that are automatically generated using machine learning techniques, as well as manual features. Our study is not limited to a specific patient group – it is instead exploring ways to make accurate predictions for patients of all age groups and with all conditions, except those admitted due to a baby birth or a psychiatric condition. We use detailed longitudinal health data from the province of Alberta, Canada. Alberta has a publicly funded, universally accessible, integrated health system and thus collects high-quality data. Our data set contains very few missing records, in particular, there exist no missing readmissions except for those who moved out of the province during the study period and those who died without being readmitted.

## Methods

### Study design

This is a population-based cohort study. We trained prediction models using linked administrative observational data from Alberta, Canada.

### Data and target population

Our target population consists of patients who were discharged from any of the acute facilities in the province of Alberta between January 1, 2015 and December 31, 2016, excluding only patients who were hospitalized due to a baby birth or a psychiatric condition. For each patient, we extracted detailed health records from 2011 to 2017 including hospitalizations from Discharge Abstract Database (DAD), ambulatory visits from National Ambulatory Care Reporting System (NACRS), physician office visits from claims data, drug prescriptions from Pharmaceutical Information Network (PIN), and lab test results.

From DAD, we extracted institution number, admit and discharge dates, discharge disposition, diagnosis codes, procedure codes, and the role of the providers associated with the patient’s care for each hospitalization. From NACRS, we obtained institution number, visit mode, visit date, disposition, diagnosis codes, procedure codes, and functional centre account code for each ambulatory visit. From claims data, we extracted information about visits to primary care physicians (family medicine), internal medicine specialists, and general surgery specialists. For each visit, we obtained the date, diagnosis codes, procedure codes, paid amount, and service provider skill code. From PIN, we extracted the following variables for each prescription: Canadian Drug Identification Number, Anatomical Therapeutic Chemical (ATC) code, date, dispensed quantity, and the number of days the prescription covers. From lab data, we received test code, test name, date, reference range, result, and unit of measure for each lab test. We extracted the lab data variables only for the lab tests listed in Appendix A in the Supplementary Material. Lastly, the extracted data also included sex, age, and the first three alpha-numerics of postal code. All diagnosis codes were ICD-10-CA except those in claims, which are ICD-9. All procedure codes are following the Canadian Classification of Health Interventions (CCI) except those in claims which were the Health Service Canadian Classification of Procedures Extended Code (CCPX).

The data were extracted and anonymized by the Alberta Strategy for Patient Oriented Research SUPPORT Unit. This study was approved by the Health Research Ethics Board of the University of Alberta (Study ID Pro00082041).

### Definition of index hospitalization

Patients may have been discharged multiple times in 2015 and 2016. We selected one index hospitalization for each patient from these years, using the following procedure. Among the discharge records (DAD) of the target population in 2015 and 2016, we first excluded from the index hospitalization selection those records whose patient died during the hospitalization or that had an invalid patient identifier, as well as those patients who had at least one record whose postal code is not in Alberta (these criteria excluded 42,900 DAD records). We further removed records whose discharge disposition indicated transfers, which excluded 42,172 DAD records. As previously mentioned, discharge records related to a birth (or with disposition indicating stillbirth or organ/tissue retrieval) were not included in the initial data extraction. We also excluded psychiatric admissions from our selection by removing records whose primary diagnosis code was related to mental and behavioural disorders (ICD F00-F99 except F10-F19), leading us to remove the 74,618 records and 18,170 patients who had only psychiatric admissions. We then randomly selected one record of each patient as the index admission and we predicted 30-day readmission after the discharge from the index admission. To define the care episode of the index hospitalization, we connected DAD records that are considered continuation of care, by using the criteria described in Appendix A. From the list of patients with an index admission (n = 428,669), we randomly divided the data into 11 equal parts. One of these was selected for the holdout test set. The remaining 10 pieces were used to perform 10-fold cross-validation for comparing models.

### Definitions of outcome and manual features

Our outcome was all-cause readmission within 30 days after discharge. In addition to the four raw features (age at discharge, sex, discharge disposition, and length of stay of the index episode -- included as part of model “manual” features), we also considered two sets of input features: derived manual ones and those automatically generated using machine learning. We first explain the derived manual features.

We computed the number of discharges and the total number of days the patient stayed in-hospital in the 6 months and 2 years prior to the current visit. The Charlson Comorbidity Index [31] was calculated based on ICD-10-CA codes of each patient’s DAD records over the past two years (including from the index admission). We also used the number of unique ICD-10-CA codes that appeared in the index episode, as well as the number of unique and total procedures performed during the index episode. We computed the numbers of emergency department (ED) visits and non-emergency outpatient visits in the past 6 months and in the past 2 years, and a binary variable if the index admission was through the ED. We also obtained the numbers of physician visits in the past 6 months and 1 year, separately for family physicians, internal medicine specialists, and general surgery specialists. As a proxy of access to care, we included binary variables whether a patient incurred a claim during the past 2, 3, and 4 years. Additionally, we calculated the total claimed dollar amounts from physician visits during the past 2, 3, and 4 years. Regarding prescriptions, we computed the number of prescription records, the total prescribed days, and the number of unique drugs (in ATC code) in the past two years and during the index episode. Features based on the twenty most common lab tests were additionally created (Appendix A). Lastly, we identified the presence of four chronic conditions (asthma, hypertension, chronic heart failure, and diabetes) using algorithms validated by Tonelli et al. [32] that use ICD9-CM/ICD10 codes. We extracted income, employment, housing status, citizenship status, and education level of the first three digits of the postal code of each patient from the 2020 Canadian Census of Population dataset [33] using the Postal Code Conversion File [34], but later removed these features as they did not improve model performance.

### Machine learned features

In addition to the manual features, we extracted feature vectors using machine learning techniques from longitudinal health records of each patient, which cover at least four years prior to their index admissions and originate from various data sources. The number of these records (a proxy for healthcare usage) varies considerably between patients. In this paper, we use *Word2Vec* [35] (from Python’s NLTK library, specifically, the Continuous Bag-of-Words implementation), an unsupervised technique borrowed from natural language processing, to encode the longitudinal information. Word2Vec not only summarizes, but also enriches the data by encoding related concepts from different data sources (e.g., a diagnosis code and a related medication) as similar numeric vectors rather than treating them as incomparable. In this process, we first created patient “sentences”, formed by collecting all medical data entries (ICD codes, ATC codes, codes representing different events such as an emergent admission, etc.) associated with a patient and sorting them chronologically. In the sentence, each word is a diagnosis code or a procedure code or an ATC code, etc. For example, if a patient accrued “K65”, “1.SQ.52”, and “J01DH” in this order in the data, their associated sentence would be “K65 1SQ52 J01DH”. More details are available in Appendix B. The Word2Vec learner then effectively creates fixed-length numeric representations of each word (medical code) based on the context within the sentences. Roughly speaking, words that tend to appear together in proximity in sentences receive numeric representations that are close. Once an inventory of numeric representations of those words has been created, each patient’s sentence can be viewed as a list of numeric vectors. There are many techniques to create a patient’s feature vector with respect to their medical history. Here, we use a simple summation of the last 25 vectors (as well as 15 for the purposes of sensitivity analysis, see Appendix C for these results as well as other values tested). If two patients have the exact same set of 25 most recent medical codes in sequence, then their resulting feature vectors are the same.

### Model training

We considered learning both logistic regression (LR) and gradient boosting machine (GBM) models for predicting readmission from our patient representations. GBMs encompass ensemble learning techniques that use many base learners, such as decision trees, to build a sequence of prediction models; and later, to predict for a novel instance, it aggregates the predicted outcomes from those individual base models. We note that our main objective is not a thorough comparison of machine learning models and we chose GBM as an example of machine learning models for illustration. GBM has parameters adjusting its training process and we used the default setting of the Python library scikit-learn [36] in all of our comparisons. In addition, we used a set of manually selected training parameters that are expected to lead to a better performance [37], to observe the impact of training parameters (called GBM Tuned). The manually selected parameters were: *learning_rate* = 0.01, *max_depth* = 8, and *n_estimators* = 1000. For definitions of these parameters, please see the scikit-learn GBM documentation [38]. In addition, an LR model based on the LACE score [39] was evaluated as a baseline. The LACE Score was developed and validated using the Canadian Discharge Abstract Database and has been externally validated [40] and become the industry standard for readmission risk prediction, which was our motivation to test LACE as a baseline in this study. All classification models described above were implemented in Python using *scikit-learn*.

## Results

### Descriptive statistics

We used data from 428,669 patients (62% female, 38% male, 27% 65 years or older, Table 1) for training and evaluating models: 24,974 (5.72%) were readmitted within 30 days of discharge for any reason. Table 1 contains summary statistics for the raw and derived manual features used, excluding lab-based features. Lab test features were not shown in the table as each of the 20 included lab tests were formatted as multiple categorical variables with many possible values (Appendix A), thus their summary is extensive. The average LACE score was found to be 7.10 (Std.Dev 3.27) over the entire study population, 6.99 (Std.Dev 3.22) for patients without a 30-day readmission event, and 8.91 (Std.Dev 3.61) for those who were readmitted in 30 days. According to Table 1, infants, seniors, and patients with hypertension or heart failure appear to have a higher chance of 30-day readmission.

**Table 1:** Raw and Manually Derived feature descriptive statistics of the whole target population, those who were not readmitted within 30 days, and those who were. The lab test features are not shown

Descriptive Statistics of Raw and Manually Defined Features			
	**All** **(n = 428,669)**	**Not readmitted in 30 days** **(n = 403,695)**	**Readmitted in 30 days** **(n = 24,974)**
**Variable**	Number (%) or Mean (Std.Dev)	Number (%) or Mean (Std.Dev)	Number (%) or Mean (Std.Dev)
Raw Features			
Sex (Female)	266,549 (62%)	253,429 (63%)	13,120 (52%)
Age			
< 1	12,841 (3%)	10,257 (3%)	2584 (10%)
1–14	20,020 (5%)	19,279 (5%)	741 (3%)
15–24	34,125 (8%)	32,931 (8%)	1194 (5%)
25–64	243,808 (57%)	233,650 (58%)	10,158 (41%)
≥ 65	117,875 (27%)	107,578 (27%)	10,097 (41%)
Length of Index Admission Stay	2 (4)	2 (4)	4 (7)
Discharge Disposition			
01: Transferred to an acute care inpatient institution	133 (0.03%)	101 (0.03%)	32 (0.1%)
02: Transferred to Continuing Care	10,081 (2%)	9311 (2%)	770 (3%)
03: Transferred to Other Facility	2249 (0.5%)	2055 (0.5%)	194 (0.8%)
04: Discharged to home or home setting with support services	33,954 (8%)	29,805 (7%)	4149 (17%)
05: Discharged home (no support services required)	376,625 (88%)	357,453 (89%)	19,172 (77%)
06: Signout (left against medical advice) & AWOL (absent without leave)	4699 (1%)	4065 (1%)	634 (3%)
07: Died	567 (0.1%)	566 (0.1%)	1 (0.004%)
12: Patients who do not return	361 (0.08%)	339 (0.08%)	22 (0.09%)
Manually Defined Features (Excluding Lab-Based Features)			
Admitted through ED - Index	377,112 (88%)	353,341 (88%)	23,771 (95%)
Num. procedures - Index	1.54 (1.70)	1.57 (1.69)	1.06 (1.87)
Num. unique procedures - Index	1.53 (1.65)	1.56 (1.64)	1.04 (1.77)
Num. unique ICD codes - Index	4.32 (3.66)	4.27 (3.59)	5.16 (4.52)
Num. unique drugs - Index / Last 2y	1.47 (2.48) / 7.18 (6.73)	1.43 (2.41) / 6.99 (6.55)	2.12 (3.26) / 10.31 (8.50)
Num. prescribed days - Index / Last 2y	46.62 (125.33) / 1730.75 (2719.67)	45.01 (122.87) / 1671.71 (2680.03)	71.35 (157.82) / 2685.03 (3144.93)
Num. prescription records - Index / Last 2y	2.40 (16.83) / 53.85 (162.04)	2.33 (15.68) / 51.39 (156.83)	3.49 (29.76) / 93.62 (226.78)
Num. hospital admissions - last 6 m / 1y	0.16 (0.50) / 0.26 (0.72)	0.14 (0.45) / 0.23 (0.64)	0.46 (0.98) / 0.73 (1.43)
Num. emergency department visits - last 6 m / 1y	1.45 (2.58) / 2.06 (4.00)	1.39 (2.44) / 1.97 (3.74)	2.44 (4.18) / 3.52 (6.76)
Num. non-emergency department visits - last 6 m / 1y	3.72 (8.69) / 5.63 (14.76)	3.60 (8.30) / 5.42 (14.07)	5.52 (13.46) / 8.92 (22.96)
Total length-of-stay in hospital - last 6 m / 2y	1.47 (8.12) / 4.78 (21.28)	1.26 (7.41) / 4.26 (19.77)	4.98 (15.25) / 13.26 (37.07)
Num. General Practice Visits - last 6 m / 1y	8.98 (10.89) / 14.29 (16.77)	8.79 (10.43) / 13.97 (16.03)	12.18 (16.27) / 19.62 (25.37)
Num. General Surgery Visits - last 6 m / 1y	0.52 (2.09) / 0.68 (2.80)	0.50 (1.94) / 0.65 (2.56)	0.79 (3.73) / 1.14 (5.35)
Num. Internal Medicine Visits - last 6 m / 1y	1.00 (3.61) / 1.44 (5.22)	0.92 (3.28) / 1.32 (4.71)	2.26 (6.98) / 3.37 (10.22)
Patient with physician claim incurred - last 2y / 3y / 4y	416,397 (97%) / 417,609 (97%) / 418,188 (98%)	393,196 (97%) / 394,359 (98%) / 394,921 (98%)	23,201 (93%) / 23,250 (93%) / 23,267 (93%)
Claimed amount - last 2y / 3y / 4y	1835.68 (2038.73) / 2389.30 (2601.73) / 2870.56 (3086.34)	1790.39 (1932.89) / 2330.13 (2470.42) / 2800.01 (2934.25)	2567.71 (3222.20) / 3345.84 (4069.96) / 4010.92 (4790.24)
Comorbidity			
Charlson score, mean (Std.Dev)	0.42 (0.99)	0.40 (0.96)	0.81 (1.42)
Hypertension	124,407 (29%)	114,068 (28%)	10,339 (41%)
Diabetes	59,359 (14%)	54,119 (13%)	5240 (21%)
Heart failure	26,217 (6%)	22,534 (6%)	3683 (15%)
Asthma	8503 (2%)	8013 (2%)	490 (2%)

**Fig. 1:**
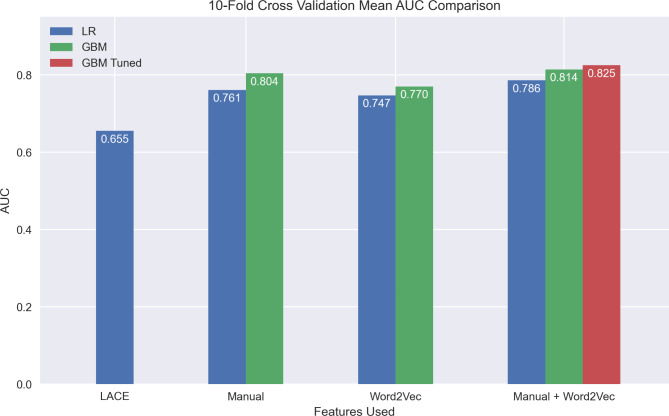
Average Area Under the Curve (AUC) comparison for Logistic Regression (LR) and Gradient Boosting Machines (GBM) on different feature sets using 10-fold cross validation. See the ‘Methods - Model Training’ section for an explanation of the model GBM Tuned

**Fig. 2:**
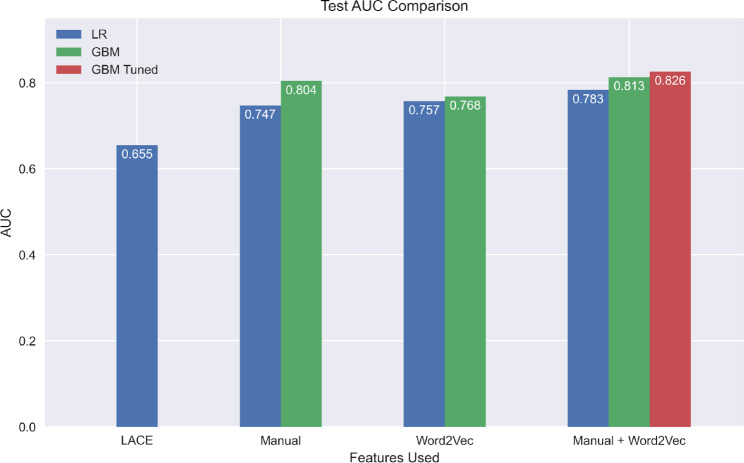
Area Under the Curve (AUC) comparison for Logistic Regression (LR) and Gradient Boosting Machines (GBM) on the test data using different feature sets. See the ‘Methods - Model Training’ section for an explanation of the model GBM Tuned

Table 2 shows AUC scores of different models evaluated by 10-fold cross-validation (Fig. 1) and using the test set, which was not used during the model building (Fig. 2). It also reports the standard deviation of the training-set AUC from cross-validation. Here, we compare the performance of LR and GBM, each trained with different combinations of manual and Word2Vec features. In Word2Vec, all results used the last 25 codes (this value outperformed other candidates such as 15 codes, see Appendix C) in the patient vector summation step. Other sensitivity analyses can be found in Appendix C.

**Table 2:** Area Under the Curve (AUC) of models generated with Python, where the Word2Vec features are the sum of the numeric vectors of the last 25 codes

Validation and Test AUCs of Different Models and Feature Configurations			
**Model**	**Features**	**Average Cross-Validation AUC (St. Dev.)**	**Test AUC**
Logistic Regression (LR)	Manual	0.7612 (0.004123)	0.747
	Word2Vec	0.7470 (0.005600)	0.757
	Manual and Word2Vec	0.7862 (0.005758)	0.783
Gradient Boosting Machine (GBM)	Manual	0.8037 (0.004001)	0.804
	Word2Vec	0.7700 (0.005138)	0.768
	Manual and Word2Vec	0.8138 (0.004534)	0.813
	Manual and Word2Vec^†^	0.8249 (0.004549)	0.826
Logistic Regression (LR)	LACE	0.6548 (0.006444)	0.655

^†^GBM with manually selected training parameters (see Section Methods - Model Training)

**Fig. 3:**
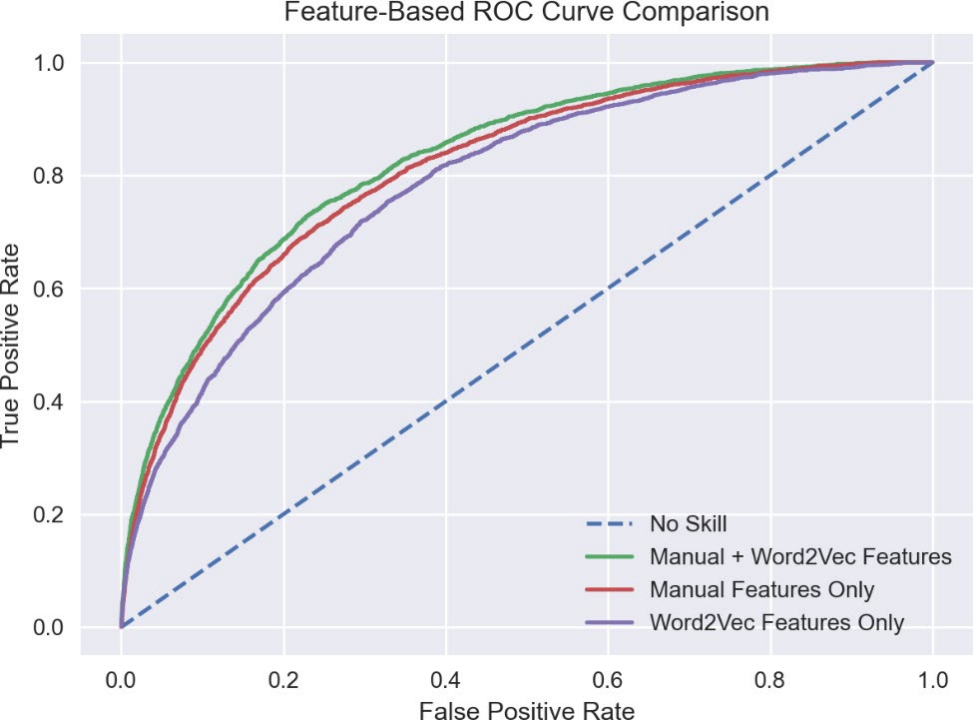
Receiver Operating Characteristic (ROC) Curves for Gradient Boosting Machine (GBM) models trained using only Word2vec features, only manual features, and a combination of Manual and Word2Vec features

Using both manual and Word2Vec features in combination yields the best results regardless of the model used (LR: 0.786 ± 0.0058, GBM: 0.814 ± 0.0045, GBM with tuned parameters: 0.825 ± 0.0045, all from cross-validation; see Fig. 3 for ROC curves). Each of these comparisons is statistically significant after Bonferroni correction using paired t-tests, P < 0.00001. Within LR, Word2Vec features alone perform the second-best with a test AUC of 0.757, followed by manual features alone with an AUC of 0.747. Within GBM, the manual feature model yielded a test AUC of 0.804. The Word2Vec model yielded a test AUC of 0.768. We compared the sensitivity of the GBM models trained on the three different feature sets when the specificity is fixed at 0.75. The sensitivity of the Word2Vec features was 0.653, the manual features yielded 0.716, and the combination of the two was 0.748. In addition, we computed the net reclassification improvement (NRI) [41] from the GBM model with manual features to the GBM model with both manual and Word2Vec features. The NRI was 0.0142 with a 95% confidence interval (CI) [0.0006, 0.0278]. The NRI for events (readmission) was 0.0059 with a 95% CI [-0.0073, 0.0191], and the NRI for non-events was 0.0083 with a 95% CI [0.0050, 0.0116]. The LR LACE baseline far underperformed the rest of our models with AUCs of 0.655 from cross-validation and 0.655 on the test set. Though our main purpose is to compare feature sets, the results also provide a comparison between models. Fixing the set of features used, GBMs perform better than LR models. When using manual and Word2Vec features, GBM is statistically better during cross-validation than LR according to a two-sided paired t-test with P < 0.001. We computed the NRI between the two models, both with manual and Word2Vec features. The NRI was 0.0570 with a 95% CI [0.0407, 0.0732]. The NRI for events was 0.0354 with a 95% CI [0.0197, 0.0511], and the NRI for non-events was 0.0216 with a 95% CI [0.0177, 0.0255]. We also evaluated the performance of our best model (GBM Tuned) on different subpopulations and obtained the feature importance analysis result of the model. These results can be found in Appendix D.

## Discussion

In this paper, we built features and models to predict 30-day readmissions using seven years of data from 429 K patients. We considered Word2Vec features, which were automatically generated using machine learning techniques, as well as manual features. Our analysis shows that Word2Vec features improve the prediction accuracy and that equipping an advanced prediction model with both the manual and Word2Vec features achieves the best performance. Our best model achieved an AUC of 0.83 on a test set over 42 K patients, which was not used during the model building.

Using only the automated features also showed good performance. LR using only the Word2Vec features had AUC 0.76 and GBM using the same features was AUC 0.77. This shows the potential of using features that are automatically generated without domain experts’ manual work. We also note that using only *manual features* performed well, too: LR (resp., GBM) achieved AUC 0.75 (resp., 0.80), which is similar to or higher than most of the models reported in literature. This underscores a major strength of this study – the high quality of the data used, which likely contributed to the high AUC values obtained using manual features. Regarding data quality, the province of Alberta has a single payor, universally accessible, integrated health system, which enables the collection of comprehensive administrative data, with minimal loss to follow-up. In all the feature combinations, GBM consistently performed better than LR. This showcases the importance of utilizing more recent advancements in machine learning to make better predictions in the health sector; it is likely that even higher performance could be achieved by employing and tuning state-of-the-art classification techniques, at the cost of a higher computational load. Another strength is that our model makes predictions for all age ranges, covers both medical and surgical admissions, and is not limited to patients with a specific condition.

Our study is not without limitations. First, linked administrative data were used, which are a less complete and less detailed data source compared to electronic medical or electronic health records. Accordingly, information from the latter type of repository, such as narrative physician and allied health notes, may further improve prediction accuracy when incorporated into the approach studied here. Second, if a patient died after discharge without being readmitted, then the death was not captured in our data. Third, although our results can be considered generalizable to other single-payor, universally accessible health systems (such as those in other Canadian provinces), generalization beyond this setting should be performed with caution. Lastly, in the process of building Word2Vec features, we added the numeric representations of words in a patient’s sentence to obtain a feature vector for the patient. More sophisticated methods to combine the numeric vectors may improve the prediction performance.

Overall, the models we created performed similarly well, but using a machine learning model along with the computer-generated features improved the prediction accuracy. Using only the Word2Vec features produced models with AUCs similar to or higher than previous work based on features automatically generated from electronic health records [19, 26]. Although the performances of different studies cannot be compared directly due to different methods and samples, these results validate the potential of the proposed automatic feature generation. There have been some attempts to define a large number of features manually from longitudinal data and apply feature selection methods [20–24, 42]. However, it is unclear how to represent temporal aspects as features (for example, one has to determine whether to distinguish the same diagnosis code issued one week ago vs. three months ago and how). Also, the manual method may be labor-intensive and less applicable generally across different systems. Our paper provides a comprehensive and automated method to derive features from longitudinal data that takes the temporal components into account. Also, there have been some studies training a deep learning model such as a convolutional neural network using longitudinal data [19, 25, 30, 43]. In contrast, we present a feature generation method that summarizes longitudinal data (including its temporal aspects) into a single feature vector so that it can be used to train any prediction model. The suggested method provides an interpretation of the generated features (Appendix B), which is often difficult in deep learning prediction models. The fact that using both kinds of features results in the best accuracy in our study raises the question of whether it is feasible to improve the automated feature generation to such an extent that the need for manual features can be eliminated.

To implement the presented model, we needed to link administrative data to create patient sentences, compute the numeric representations of the sentence components (e.g., diagnosis codes, procedure codes, etc.), and build the prediction model. Once the model is trained, we can make a prediction for a new patient by first converting his/her records into a sentence, computing the Word2Vec features of the patient (by using the numeric representations of words previously obtained), and computing the manual features for models that require them. Note that all of these steps after model training can be automated in practice and require the same data access as the LACE model, because both require accessing administrative data of a patient. The major computations of our framework are learning the numeric representations of codes and training a machine learning model, which are done *a priori*, before making a prediction for a new patient. Computing the features of the target patient in real-time would require linking the patient’s data from different sources in real-time. This study benefited from the fact that the data are from an integrated health system. However, such integrated data sets are expected to become more available in the near future (e.g., the CRISP program [44], as well as others [45–47]), and our study demonstrates the potential of those initiatives to innovate healthcare delivery. Also, we highlight that our model is not limited to a specific subpopulation and yet showed high performance. Deploying a unified model can save tremendous amounts of administrative cost and effort compared to deploying multiple models.

In general, it is more desirable to predict readmission at the time of admission than the time of discharge, but the prediction timing of most past studies is at discharge [9]. The present study predicts readmissions also at discharge because our models used some variables from the index hospitalization episode in addition to records from before the index admission. Therefore, building models that predict at the time of admission is beyond the scope of the current study. It is also preferable to predict preventable readmissions so that appropriate action could perhaps be taken to avoid the second admission. However, past studies have shown a wide variation in the definitions of preventable readmissions and, therefore, operationalizing models to predict this outcome remains a challenge. One important use of readmission prediction is to inform targeted interventions that may prevent readmissions. Past studies suggest that some post-discharge interventions can reduce readmissions and save associate costs [48, 49]. For example, Alberta provides home-based acute care for individuals with chronic or complex diseases or low acuity medical conditions. Because resources for these interventions are limited, health systems are under pressure to better target these interventions. Using our prediction model to help decisions regarding these interventions is a future direction to explore.

In conclusion, we have shown that using both computer-generated and manual features improved prediction accuracy over manually-derived features alone and over a LACE model. This demonstrates that modeling using machine learning features can improve upon conventional methods, illustrating the potential of this new method to improve understanding of readmission and its effect on clinical care delivery.

## Electronic supplementary material

Below is the link to the electronic supplementary material.

Supplementary Material 1

## Data Availability

The data that support the findings of this study are available from the Alberta Health Services but restrictions apply to the availability of these data, which were used under a data disclosure agreement for the current study, and so are not publicly available. To inquire about the data access, contact I.L. at ilbin@ualberta.ca. To request authorization to obtain data by direct access, contact research.administration@ahs.ca.
