# Supplementary material for: Effective hospital readmission prediction models using machine-learned features
Source: BMC Health Serv Res. 2022 Nov 24;22:1415. doi: 10.1186/s12913-022-08748-y (PMC9700920; doi:10.1186/s12913-022-08748-y)
Supplement: Supplementary file 1 — Supplementary Material 1 [file 12913_2022_8748_MOESM1_ESM.docx]

SUPPLEMENTARY MATERIAL

Appendix A - Data Processing

**Features from Lab Data:**

We included 20 laboratory tests in our analysis shown in the list below. For each laboratory test, we divided the numeric results into deciles of the result values, and labeled the smallest as one, the second smallest one was two, and so on, through ten. The included laboratory tests were the following.

1. White Blood Count
2. Platelet Count
3. Hemoglobin
4. LDL Cholesterol
5. HDL Cholesterol
6. Non-HDL Cholesterol
7. Creatinine
8. Thyroid Stimulating Hormone
9. Hemoglobin A1C
10. Ferritin
11. Alkaline Phosphatase
12. Prothrombin Time International Ratio
13. Albumin
14. Glucose random
15. Calcium
16. Ionized calcium
17. C-Reactive Protein
18. Bilirubin total
19. Bilirubin,direct
20. Lipase

Raw results from these lab tests, as well as features crafted using three different decile values (5, 10, 20) were used for our models that included manual features. Additionally, we experimented with incorporating these lab results in our Word2Vec model as “words” in patient sentences (Appendix C.4, Table A.3).

**Index Admission Definition:**

When selecting index hospitalizations, we considered only those DAD records whose discharge disposition was ‘transferred to continuing care’, ‘transferred to other facilities’, ‘discharged to home or home setting with support services’, ‘discharged home with no support services’, ‘sign out (left against medical advice) or absent without leave’, or ‘patients who do not return from pass’.

After selecting a DAD record as the index hospitalization for each patient, we determined the care episode of the index hospitalization as follows. If two DAD records overlapped, that is, the discharge date of one of them is after the admission date of the other, then we considered them as continuation of care regardless of their discharge dispositions. Also, if there were two DAD records where the discharge date of one of them either coincides with or is one day before the admission date of the other, then again, we considered them as continuation of care regardless of their discharge dispositions. Using these criteria, we combined DAD records starting from the randomly selected index DAD record to form *the index episode* for each patient. The index episode ends when a patient has no other DAD record on either the episode end date or the day after. Then, we predict readmissions within 30 days after the end date of the index episode. Note that all input features regarding index hospitalization (such as the number of procedures during the index episode) are computed from the whole index episode.

Appendix B - Machine Learned Features

In this appendix, we discuss in detail how the machine learning features were generated. We describe tools and concepts for generating medical concept vectors, illustrate some example outputs, and explain how we obtained a feature vector for each patient.

**Word2Vec:**

In this section, we explain the general method we employed to translate each medical code in a patients’ health history into a numeric representation. Word2Vec^1^ contains an implementation of the *continuous bag-of-words* architecture, a class of neural network models from the domain of natural language processing. It was designed to learn fixed-length numeric representations of words.

Large and/or sparse vectors for a “final” representation pose many challenges in the realm of machine learning. Word2Vec uses a neural network architecture (single-hidden-layer feed-forward) to learn a lower-dimensional representation of concepts using two higher-dimensional representations (one “One Hot Encoded” representation, and one probabilistic representation calculated from the data). When the network weights are trained to completion, the lower-dimensional representation for each word can be calculated by extracting the values at the network’s hidden nodes.

**Applying Word2Vec to Obtain Medical Concept Vectors:**

In this section we explain how the method described in the previous section is applied to this paper. First, we explain the overall approach. Consider a patient with substantial hospital usage over the last several years. During those years, this patient would have accrued medical codes, such as diagnoses and procedures, associated with the provided care. By listing these medical codes in chronological order, we now have a representation of this patient, which is analogous to a document containing a sequence of words. From this point, Word2Vec (as explained in the previous section) can be applied to find representations of medical concepts, in which similar or related codes (e.g., the ICD-9 code for asthma, the ICD-10 code for asthma, and the drug code for an asthma medicine) have similar vector representations.

Now we explain how we convert each patient’s records into a sentence. First, each ICD code becomes a word, rounded to the first three alphanumerics. Each CCI and ATC code becomes a word, where the first five characters were retained. Each CCPX code is also a word, for which one point after the decimal was kept.

Each inpatient admission in DAD is included as a word. Discharge is also a word, where we categorize discharge events according to the dispositions. Each ambulatory visit in NACRS is also a word. We distinguish emergent and non-emergent visits. We defined an emergent visit as an NACRS record whose MIS_CODE (a variable representing the type of visit) is one of the following: 713100000 (Emergency), 715130000 (Community Urgent Care Clinic), 715140000 (Community Advanced Ambulatory Care Centre), and 713102000 (General Emergency). The rest are categorized as non-emergent. Each discharge from an NACRS visit is also a word, and we also categorize the discharge events according to the dispositions.

Each lab result also became a word. We considered only those lab tests listed in Appendix A. We tested the inclusion of only these lab test names in the patient sentences (without regard to the results) as well as appending the decile labels (described in Appendix A) for each lab test to incorporate the result. For example, if creatinine level was measured for a patient and the result was the second decile, then the word ‘Creatinine2’ is added to the patient’s sentence.

Also, following other work using similar techniques^2,3^, we added codes to represent time elapsed between the codes explained above. Five words were used to denote the number of months between interventions: <1, 1-3, 3-6, 6-12, and 12+. Gaps that were smaller than seven days were not assigned any special code under the assumption that any medical codes dispensed within a week of each other are likely related. Lastly, we replaced codes with a frequency of fewer than 100 occurrences in the whole data with the term "RAREWORD".

In sum, the patient sentences contain words representing diagnosis codes (ICD-9 and ICD-10), procedures (CCI and CCPX), drugs (ATC), lab results, hospital admissions and discharges, ambulatory visits and discharges, and time gaps. The sentence of a patient is obtained by ordering these codes chronologically. In the data, we often observed long and repeating stretches of the same code (such as continued prescriptions of the same drug), which we collapsed into a single appearance of the code. This was done to ensure that the Word2Vec model could learn contextual associations between codes, as is similarly done in natural language processing^4^. In addition, since the exact order of the codes between two time gap words is often not clear (for example, diagnosis codes and procedure codes from the same visit), we shuffled codes between time gap words, following Choi et al.^3,5^. It has been shown that shuffling can decrease the bias found in Word2Vec models^6^.

The procedures explained in this section generate the patient sentences from the data, which are then fed into the Word2Vec algorithm explained in the previous section. The output is the numeric vectors representing those words in the sentences (e.g., diagnosis codes, drug codes, an admission event, etc.). In the next section, we illustrate some examples of the numeric vectors.

**Illustrative Examples of Medical Concept Vectors:**

The Word2Vec algorithms are designed so that words that have similar usages have similar numeric representations. In this section, we show some examples of the numeric vectors to illustrate how the Word2Vec algorithm worked. For each of four diagnosis codes associated with highly prevalent chronic conditions^7^ (namely diabetes, hypertension, chronic heart failure, and asthma), we found five words that are the closest in the numeric representations. The distance between numeric vectors were measured using the cosine similarity. This analysis can be considered a sanity-check to ensure that Word2Vec captures essential semantic information. The results are shown in Appendix Table A.1.

| Medical Concept Vector Interpretation for Chronic Conditions | | | | | |
| --- | --- | --- | --- | --- | --- |
| **Condition Code** | **Condition Name** | **Similar Codes** | **Cosine Similarity** | **Similar Code Type** | **Description** |
| E11 | Type 2 diabetes mellitus | E14 | 0.777 | ICD-10 | Unspecified diabetes mellitus |
|  |  | N08 | 0.752 | ICD-10 | Glomerular disorders in diseases classified elsewhere |
|  |  | I10 | 0.743 | ICD-10 | Essential (primary) hypertension |
|  |  | E78 | 0.661 | ICD-10 | Disorders of lipoprotein metabolism and other lipidemias |
|  |  | A10BX | 0.612 | ATC | Drugs used in diabetes: Other blood glucose lowering drugs |
| I10 | Essential (primary) hypertension | E11 | 0.743 | ICD-10 | Type 2 diabetes mellitus |
|  |  | E78 | 0.729 | ICD-10 | Disorders of lipoprotein metabolism and other lipidemias |
|  |  | I25 | 0.630 | ICD-10 | Chronic ischemic heart disease |
|  |  | N08 | 0.572 | ICD-10 | Glomerular disorders in diseases classified elsewhere |
|  |  | Z95 | 0.514 | ICD-10 | Presence of cardiac and vascular implants and grafts |
| J46 | Asthma | 493 | 0.722 | ICD-9 | Asthma |
|  |  | 1.GZ.35 | 0.671 | CCI | Inhalation pharmacotherapy |
|  |  | J98 | 0.608 | ICD-10 | Other respiratory disorders |
|  |  | R03AC | 0.600 | ATC | Drugs for obstructive airway disease: Selective beta-2-adrenoreceptor agonists |
|  |  | R03BA | 0.594 | ATC | Drugs for obstructive airway diseases: Glucocorticoids |
| I50 | Heart Failure | 428 | 0.863 | ICD-9 | Heart failure |
|  |  | I42 | 0.805 | ICD-10 | Cardiomyopathy |
|  |  | I25 | 0.706 | ICD-10 | Chronic ischemic heart disease |
|  |  | I48 | 0.688 | ICD-10 | Atrial fibrillation and flutter |
|  |  | I34 | 0.674 | ICD-10 | Nonrheumatic mitral valve disorders |

**Table A.1.** Five most similar codes associated with four highly prevalent chronic conditions. The cosine similarity was calculated on the automatically-generated Word2Vec vector representations

**Creating Patient Vectors:**

In this section, we explain how we obtain a feature vector for a patient using the data and the medical concept vectors obtained in the previous sections. Since we have a sentence for each patient describing the care history of the patient and a numeric representation of each word in the sentences, now we have a list of numeric vectors for each patient. In this paper, we use a simple summation of the vectors to summarize a patient’s care history and use it as a feature vector for prediction. The intuition behind this lies in an important property of Word2Vec that certain vector operations performed on the numeric vectors (e.g., addition, subtraction) are meaningful. A well-known example demonstrating this is:

vec(king) - vec(man) + vec(woman) ≈ vec(queen).

Assuming that data for training the numeric representations are representative enough, the above equation should hold for a set of Word2Vec vector representations.

When adding the concept vectors to create a patient feature vector, we used only the last *k=*15 or 25 words to capture the most recent information. Experimental results comparing the prediction performance of different *k* values can be found in our sensitivity analysis (Appendix C).

Appendix C - Additional Results

In this section, we present further results meant to supplement what is presented in the main body of this paper. The additional parameters we varied (beyond ML algorithm and inclusion of different feature sets, see main text) include (1) whether lab data are incorporated in the Word2Vec features or not, (2) programming language on which the models were built, and (3) the number of the most recent codes used in the Word2Vec feature building. Each of the experimental settings used is described below. These results may or may not statistically differ from each other but were nonetheless included to aid in future decision-making.

**Subsection 1: Choice of Machine Learning Algorithm**

We used logistic regression, gradient boosting machines, and support vector machines for our classification task due to their popularity and/or ease of use. Gradient Boosting Machines performed the best in all combinations of parameter settings. Support Vector Machines had comparable results to Gradient Boosting Machines when trained in Python but took many times as long to train in R, and were too inefficient to be feasible with the data shape and size. We additionally included the LACE baseline AUC estimated using logistic regression^8^.

**Subsection 2: Inclusion of Different Feature Sets**

We report results for three cases: (1) only using manual clinical features, (2) only using automatically generated Word2Vec features, and (3) using both manual clinical features and automatically generated Word2Vec features simultaneously. More information can be found in the results section in the main text. Please refer to Figure 3 in the main text to see a comparison of the ROC curves using the three different feature sets.

**Subsection 3: Inclusion of Lab Data in Word2Vec Model**

Further, we tested how incorporating varying amounts of information from lab data into the Word2Vec features changed the performance.  We compared the results when excluding lab data from the Word2Vec features altogether, incorporating all lab test names, incorporating only doctor-recommended lab test names, incorporating all lab test names without the lab results, and incorporating the doctor recommended lab test names with the appended token that describes the test result interval (see Appendix A for details). In our experiments, incorporating only the test names without any result information did not improve the performance. Table A.3 shows the rest of the results. We also tried varying the number of intervals to obtain the lab test result tokens from 10 to 15 and 20. Using 10 intervals performed similarly or better than 15 and 20 intervals. Overall, including the lab data in the Word2Vec features seems to be disadvantageous, therefore these lab codes were left out of the results in the main text.

**Subsection 4: Choice of Programming Language (Table A.2)**

We additionally compared the performance of these models implemented in Python versus R. To train models in R, we used the stat library for logistic regression (LR) and gbm library for GBM. In the case of LR, the performance values between Python and R are similar. However, the AUC values initially obtained through R using gradient boosting machines appeared to be higher than their Python counterparts. This was likely due to differences in default parameter settings and/or other implementation-specific details. However, after experimenting with some GBM parameters in Python (Table A.2), the results for our best experimental setting between Python and R appeared quite similar. Our final evaluation on the test set was done using Python (see table 3 in main text) tables.

| Test AUCs by Programming Language | | | |
| --- | --- | --- | --- |
| **Model** | **Features** | **Test AUC (Python)** | **Test AUC (R)** |
| Logistic Regression | Manual | 0.747 | 0.7414 |
|  | Word2Vec | 0.755 | 0.7525 |
|  | Manual and Word2Vec | 0.783 | 0.7805 |
| GBM  (Parameter Tuned) | Manual | 0.804 | 0.8147 |
|  | Word2Vec | 0.768 | 0.7884 |
|  | Manual and Word2Vec | 0.826 | 0.8276 |
| Logistic Regression | LACE | 0.655 | 0.662 |

**Table A.2**. Test Area Under the Curve (AUC) for Logistic Regression (LR) and Gradient Boosting Machines (GBM) comparing Python and R.

**Subsection 5: Choice of Number of Medical Codes (Table A.3)**

We tested many different values for the number of most recent medical codes to include in a patient feature vector (denoted by *k*), and we found that using the last 15 and 25 codes consistently outperformed the other numbers we tested (*k* = 5, 10, 15, …, 100). Table A.3 shows how these two values compare. Overall, *k* = 25 leads to the best AUC results, so this is what was used in our main results section.

| Test AUCs by Inclusion of Lab Codes and Number of Medical Codes Summed | | | | |
| --- | --- | --- | --- | --- |
| **Model** | **Features** | **Lab in Word2Vec** | ***k =* 15 codes**  **AUC (Std.Dev)** | ***k =* 25 codes**  **AUC (Std.Dev)** |
| Logistic Regression | Manual | N/A | 0.7612 (0.004123) | |
|  | Word2Vec | No | 0.7545 (0.005296) | 0.7470 (0.005600) |
|  |  | Yes | 0.7473 (0.005984) | 0.7603 (0.005917) |
|  | Manual and Word2Vec | No | 0.7847 (0.005216) | 0.7862 (0.005758) |
|  |  | Yes | 0.7818 (0.005212) | 0.7864 (0.006391) |
| GBM | Manual | N/A | 0.8037 (0.004001) | |
|  | Word2Vec | No | 0.7669 (0.005205) | 0.7700 (0.005138) |
|  |  | Yes | 0.7552 (0.004308) | 0.7652 (0.005793) |
|  | Manual and Word2Vec | No | 0.8126 (0.005219) | 0.8138 (0.004534) |
|  |  | Yes | 0.8109 (0.005243) | 0.8122 (0.005776) |
| Logistic Regression | LACE | N/A | 0.6548 (0.006444) | |

**Table A.3**. Cross-validation Area Under the Curve (AUC) for Logistic Regression (LR) and Gradient Boosting Machines (GBM) and different feature sets with and without lab codes and varying *k* in Python. The label “No” in the “Lab in Word2Vec” column implies that lab test codes were foregone entirely in Word2Vec training, and “Yes” means that we incorporated the physician-recommended lab codes using the 10 interval result tokens (for more details, see Appendix A).

Appendix D - Model Investigation

**Feature Importance Analysis**

The following are the ten most important features (and the associated scores) from our best-performing model, GBM tuned, when both the manual features and the Word2Vec features were used. The feature importance analysis was done using the scikit-learn^9^ library in Python. Eight of these features are either raw or manually engineered, and two come from the Word2Vec features.

1. Length of index admission stay (0.098)
2. Number of unique procedures in index admission (0.057)
3. Total number of procedures in index admission (0.050)
4. Total length-of-stay in hospital in previous two years (0.040)
5. Word2Vec element 98 (0.029)
6. Word2Vec element 45 (0.025)
7. Emergency department visits in previous six months (0.022)
8. Age at discharge (0.020)
9. Charlson comorbidity index (0.017)
10. Claimed amount in previous four years (0.014)

Since the training of the Word2Vec representations combined with the subsequent summation step involves creating a dense numeric representation of a patient’s history, the meaning of a particular Word2Vec element (e.g. the elements #98 and #45) is hard to parse. In Appendix B, we discuss interpretation of the Word2Vec representations of some codes to show that the numeric representations of related words are close to each other. However, we note that these interpretations are for each word, not the eventual patient features, and that the features are the sum of the numeric representations of words in recent medical history.

**Model Performance on Subpopulations**

Our best model (GBM Tuned) was additionally evaluated on specific subsets of the study population, grouped based on sex and age. The results are below in Tables A.4 and A.5.

| Performance of GBM Tuned by Sex | | |
| --- | --- | --- |
| Sex | Test Set Size | AUC on Test Set |
| F | 24,282 | 0.847 |
| M | 14,684 | 0.783 |

**Table A.4**. Sex-wise performance of the Gradient Boosting Machine (GBM) Tuned model, evaluated on the test set.

| Performance of GBM Tuned by Age | | |
| --- | --- | --- |
| Age Range | Test Set Size | AUC on Test Set |
| <1 | 1185 | 0.861 |
| 1 - 14 | 1830 | 0.759 |
| 15 - 24 | 3108 | 0.841 |
| 25 - 64 | 22,083 | 0.839 |
| ≥ 65 | 10,763 | 0.730 |

**Table A.5**. Age-wise performance of the Gradient Boosting Machine (GBM) Tuned model, evaluated on the test set.

Although our model does not perform poorly in any of the subpopulations, we observe that our model performs favourably for females, those under the age of 1, and for individuals aged between 15 and 64. Further investigation into the performance for individuals aged 65 and above may indicate a future research direction.

SUPPLEMENTARY MATERIAL BIBLIOGRAPHY

1. Mikolov T, Chen K, Corrado G, Dean J. Efficient Estimation of Word Representations in Vector Space. In: *ICLR*; 2013.

2. Nguyen P, Tran T, Wickramasinghe N, Venkatesh S. Deepr: A Convolutional Net for Medical Records. *IEEE J Biomed Health Inform*. 2016;PP. doi:10.1109/JBHI.2016.2633963

3. Choi E, Schuetz A, Stewart WF, Sun J. Using recurrent neural network models for early detection of heart failure onset. *J Am Med Inform Assoc JAMIA*. 2017;24(2):361-370. doi:10.1093/jamia/ocw112

4. Lilleberg J, Zhu Y, Zhang Y. Support vector machines and Word2vec for text classification with semantic features. *2015 IEEE 14th Int Conf Cogn Inform Cogn Comput ICCICC*. Published online 2015. doi:10.1109/ICCI-CC.2015.7259377

5. Choi E, Schuetz A, Stewart W, Sun J. Medical Concept Representation Learning from Electronic Health Records and its Application on Heart Failure Prediction. *ArXiv*. Published online 2016.

6. Lai S, Liu K, He S, Zhao J. How to Generate a Good Word Embedding. *IEEE Intell Syst*. Published online 2016. doi:10.1109/MIS.2016.45

7. Tonelli M, Wiebe N, Fortin M, et al. Methods for identifying 30 chronic conditions: application to administrative data. *BMC Med Inform Decis Mak*. 2015;15(1):31. doi:10.1186/s12911-015-0155-5

8. Damery S, Combes G. Evaluating the predictive strength of the LACE index in identifying patients at high risk of hospital readmission following an inpatient episode: A retrospective cohort study. *BMJ Open*. 2017;7:e016921. doi:10.1136/bmjopen-2017-016921

9. Pedregosa F, Varoquaux G, Gramfort A, et al. Scikit-learn: Machine Learning in Python. *J Mach Learn Res*. 2011;12(85):2825-2830.
